# Oral Microbiome: Getting to Know and Befriend Neighbors, a Biological Approach

**DOI:** 10.3390/biomedicines10030671

**Published:** 2022-03-14

**Authors:** Cecilia Bacali, Romana Vulturar, Smaranda Buduru, Angela Cozma, Adriana Fodor, Adina Chiș, Ondine Lucaciu, Laura Damian, Mirela Liliana Moldovan

**Affiliations:** 1 Department of Prosthodontics and Dental Materials, “Iuliu Hatieganu” University of Medicine and Pharmacy, 32 Clinicilor St., 400006 Cluj-Napoca, Romania; cecilia.m.bacali@gmail.com (C.B.); dana.buduru@umfcluj.ro (S.B.); 2 Department of Molecular Sciences, “Iuliu Hațieganu” University of Medicine and Pharmacy Cluj-Napoca, 6 Pasteur St., 400349 Cluj-Napoca, Romania; adinachis82@gmail.com; 3 Cognitive Neuroscience Laboratory, University Babes-Bolyai, 30 Fântânele St., 400294 Cluj-Napoca, Romania; 4 4th Medical Department, University of Medicine and Pharmacy “Iuliu Hatieganu” Cluj-Napoca, 18 Republicii St., 400015 Cluj-Napoca, Romania; angela.cozma@umfcluj.ro; 5 Clinical Center of Diabetes, Nutrition and Metabolic Diseases, “Iuliu Hatieganu” University of Medicine and Pharmacy, 2-4 Clinicilor St., 400012 Cluj-Napoca, Romania; adriana.fodor@umfcluj.ro; 6 Department of Oral Health, University of Medicine and Pharmacy “Iuliu Hatieganu”, 400012 Cluj-Napoca, Romania; patricia.lucaciu@umfcluj.ro; 7 Department of Rheumatology, Emergency Clinical County Hospital Cluj, Centre for Rare Autoimmune and Autoinflammatory Diseases, 2-4 Clinicilor St., 400006 Cluj-Napoca, Romania; ldamian.reumatologie@gmail.com; 8 CMI Reumatologie Dr. Damian, 6-8 Petru Maior St., 400002 Cluj-Napoca, Romania; 9 Department of Dermopharmacy and Cosmetics, Faculty of Pharmacy, “Iuliu Hatieganu” University of Medicine and Pharmacy, 12, I. Creanga St., 400010 Cluj-Napoca, Romania; mmoldovan@umfcluj.ro

**Keywords:** biofilm, metabolomics, oral diseases, systemic diseases, genomics, Oral Microbiome Database, amyloid, immune responses, autoimmune diseases

## Abstract

The oral microbiome, forming a biofilm that covers the oral structures, contains a high number of microorganisms. Biofilm formation starts from the salivary pellicle that allows bacterial adhesion–colonization–proliferation, co-aggregation and biofilm maturation in a complex microbial community. There is a constant bidirectional crosstalk between human host and its oral microbiome. The paper presents the fundamentals regarding the oral microbiome and its relationship to modulator factors, oral and systemic health. The modern studies of oral microorganisms and relationships with the host benefits are based on genomics, transcriptomics, proteomics and metabolomics. Pharmaceuticals such as antimicrobials, prebiotics, probiotics, surface active or abrasive agents and plant-derived ingredients may influence the oral microbiome. Many studies found associations between oral dysbiosis and systemic disorders, including autoimmune diseases, cardiovascular, diabetes, cancers and neurodegenerative disorders. We outline the general and individual factors influencing the host–microbial balance and the possibility to use the analysis of the oral microbiome in prevention, diagnosis and treatment in personalized medicine. Future therapies should take in account the restoration of the normal symbiotic relation with the oral microbiome.

## 1. Introduction

**Figure 1:**
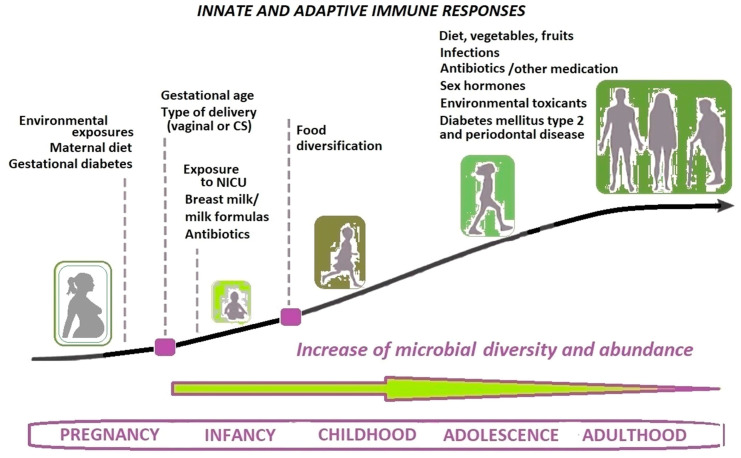
The main factors influencing the evolvement of human microbiome over the lifespan (adapted after [5,6,13]). NICU—neonatal intensive care unit; CS—cesarean section.

Animal bodies are host for various symbiotic microbial species, forming a complex association throughout the organism’s lifetime [1,2,3]. Bacteria were involved in animal bodies’ functions for more than 500 million years, having evolved together ever since [4]. The host factors can positively affect the microbiome, promoting balance and diversity between different types of species, resulting in a state of symbiosis and absence of pathology [5]. Moreover, co-evolution has led to interdependence: the human microbiome influences a large array of essential functions of the host, affecting a variety of physiologic, immunologic and metabolic processes, including the training and development of the host’s innate and adaptive immune system [6]. The term of microbiome, first introduced by Lederberg in 1958, signifies the ecological community of commensal, symbiotic and pathogenic microorganisms that share our body space [7]. The definition of microbiome is more complex than initially considered, encompassing, besides bacteriome, fungi (mycobiome) virus (virome) and ultrasmall organisms (candidate phyla radiation group) [8,9]. In the meantime, the host provides its microbes with an appropriate environment for their maturation and growth [5]. The human microbiome has an important body-site specificity: the oral microbiome has distinct patterns of composition and function from the microbiome of the skin, vagina or the distal gut [6]. Species or strains also vary according to the diet, age, use of antibiotics, health status, genetics, environmental exposures (to xenobiotics or microorganisms), disease state, socioeconomic status, geography and pregnancy status [6,10,11,12] (Figure 1).

The perturbations in the size and composition of a specific microorganism’s communities have been involved in a large array of pathology: metabolic, gastrointestinal, hepatic, neurologic, autoimmune, oncologic, cardiovascular or even in psychology [6,7]. 

The contribution of human microbiome to disease susceptibility and to the pathogenesis and progression of systemic diseases is only beginning to be understood.

## 2. Modern Methods for Studying the Human Microbiome

Microbiome studying was limited until recently to the conventional culture-dependent procedures; nevertheless, the impossibility of studying all of the microbes in isolation makes the microbial investigation more difficult [14]. Nowadays, the Human Oral Microbiome Database (HOMD) [http://www.homd.org, accessed in January 2022] provides for the scientific community comprehensive information on the predominant bacterial species identifiable in the human oral cavity; this database contains the information of over 770 prokaryotic species [15,16]. 

**Figure 2:**
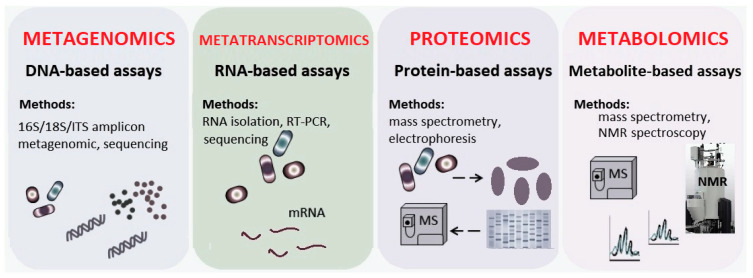
Representation of molecular approaches for modern studies host–microbiome interactions. Several aspects of the *Central Dogma of Molecular Biology*—illustrating the flow of genetic information from DNA to mRNA to protein—can be assessed to study host–microorganism and microorganism–microorganism interactions at the molecular level in human populations (adapted after [6,18,19]). Legend: mRNA: messenger ribonucleic acids, ITS: Internal Transcribed Spacer, RT-PCR: Reverse Transcription—Polymerase Chain Reaction, MS: mass spectrometry, NMR spectroscopy: Nuclear Magnetic Resonance spectroscopy.

Current methods to study the oral microbiome include cultures and microscopy, gel-based techniques, polymerase chain reactions methods, DNA microarrays and Next Generation Sequencing (NGS) techniques as part of the Human Microbiome Project that enabled a better understanding of how the microbiome impacts human health and disease [16,17]. The modern approach is based on 16S/18S/ITS amplicons sequencing. The 16s rRNA gene, a highly conserved component, is the most widely used gene marker for the identification of genus and species, and thus for taxonomic significance in bacteria and archaea. Besides, 18S rRNA investigation is used in fungi for phylogenetics. In addition, the ITS (Internal Transcribed Spacer) region (including 5.8S), a universal fungi marker, is more appropriate for analyzing intra-specific genetic marker diversity in fungi. The complexity of the microbiome has resulted in genomic and metabolomics databases for which much information remains to be annotated, including chemical source (host versus microbe), chemical structures and metabolic pathways. Large-scale efforts for generation and integration of the data will be necessary to develop computational models. Modern technologies (Figure 2) readily support small molecular proteomic and metabolite surveys (targeted or untargeted) and nucleotide sequencing of RNA and DNA to assess host and microbial gene expression, taxonomic profiles and genomes [3,6,18]:

(a) Metagenomics assays are answering the question “*what microorganisms are there and what can they do*?” and are referring to all genomes or genes encoded by a microbiota.
(b) Metatranscriptomics approaches are designed for answering the question, “*how do microorganisms respond and what pathways are activated?*”
(c) Metaproteomics approach is used to answer, “*what interactions are between microorganisms and the host and what proteins are being produced*?”
(d) Metabolomics approach for answering the question, “*what are the chemical results of their activity*?” These approaches can predict microbiome–chemical interactions and their consequences.

## 3. The Microbiome of the Oral Cavity

### 3.1. Overview

The oral cavity hosts a large number of microorganisms, the totality of them being known as the oral microbiome, the oral flora or the oral microbiota [15]. The mouth offers a favorable habitat—appropriate humidity, temperature (37 °C) and pH (6.75–7.25) and abundant nutrients for various microbial species such as bacteria, protozoa, fungi and viruses [5]. The oral cavity, though sterile at birth, contaminates with pioneer species quickly after birth, becoming the habitat for many (more than 700) microbial species during one’s lifetime, being the second most heavily colonized part of the human body [16]. Delivery mode (vaginal or through cesarean section) [20] as well as the type of feeding (breast fed or formula fed) [21] can also influence the oral microbiome. The bidirectional, indivisible relationship between the human host and its oral microbiome, evolutionarily shaped, is a constant crosstalk. Oral flora performs physiological, metabolic, immunological, mucosal protector, nutritional and also detoxifying roles [22]. 

**Figure 3:**
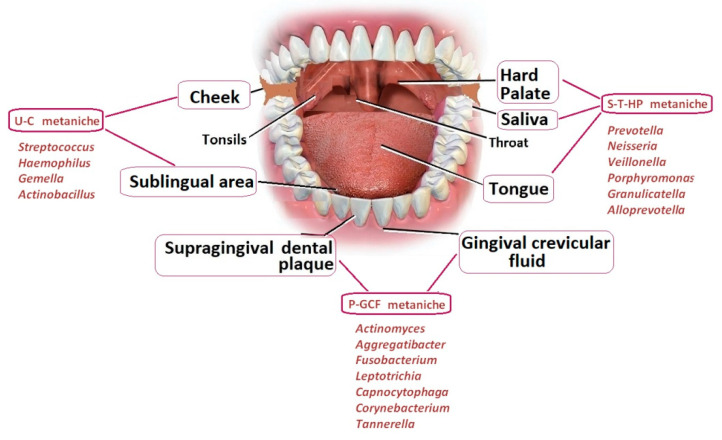
The types of biological samples that have been collected during the Human Microbiome Project population: saliva, palate, tonsils, throat, buccal mucosa (cheek), tongue soft tissues, supragingival dental plaque, etc. (adapted after [15,27]; metaniches and the composition of the oral microbiota associated with anatomically diverse oral regions: U-C metaniche: sublingual-cheek region, P-GCF metaniche: supragingival dental plaque—gingival crevicular fluid region, S-T-HP metaniche: saliva–tongue–hard palate region [28].

The oral microorganisms have been studied in different oral habitats: gingival sulcus, tongue, cheek, hard and soft palate, floor of the mouth, throat, saliva and teeth [15] (Figure 3). Some of the oral microorganisms have been found in all oral sites, while others showed site specificity [23,24]. A core microbiome common to all individuals as well as a variable microbiome, unique to each individual, depending on genetic and environmental factors [25], were described.

A change in the microbiome composition or a higher number of certain microorganisms, called dysbiosis, can be associated with certain oral or systemic diseases [26].

The Human Microbiome Project (HMP) [http://www.hmpdacc.org, accessed in January 2022] was launched in 2007 by the National Institute of Health (NIH) and International Human Microbiota Consortium (IHMC) to enable large characterization of the human microbiota and investigation of their role in human health and disease [27]. The HMP studies (based upon 4788 specimens from 242 screened and phenotyped adults) have shown that oral cavity taxon may be highly personalized. The studies also revealed, despite microbial carriage variation between subjects (concerning the species and strain level), stability of metabolic pathways in healthy population [27].

The 16S rDNA profiling of the healthy oral cavity categorized the inhabitant bacteria into six broad phyla (*Firmicutes*, *Actinobacteria*, *Proteobacteria*, *Fusobacteria*, *Bacteroidetes* and *Spirochaetes*) accounting for 96% of total oral bacteria. These oral microorganisms exhibit a direct influence on human health, from host’s metabolism to immune responses [29].

In the mouth there are several niches, local microenvironments inhabited by different microbial communities, such as supra- and subgingival plaque on teeth, gingival sulcus, hard palate, tongue, sublingual area and cheek oral area [28]. In the oral cavity, most habitats were dominated by *Streptococcus*, but these were followed in abundance by *Haemophilus* in the buccal mucosa, *Actinomyces* in the supragingival plaque and *Prevotella* in the subgingival plaque. In saliva, the main species found were *Streptococcus*, *Prevotella*, *Veillonella*, *Neisseria* and *Haemophilus* [30]. Moreover, based upon the cluster analysis of microbial genera distribution in different oral niches, three “metaniches” with similar composition were described [28] (Figure 3).

Other non-bacterial components of the healthy oral microbiota are fungi such as *Candida* spp., *Cladosporium*, *Saccharomyces*, *Aspergillus*, *Fusarium*, *Cryptococcus* spp. and others, the latter associated with increased infection risk [30,31]. Saprophytic protozoa such as *Entameba gingivalis* and *Trichomonas tenax*, as well as Archaea have been reported as well [31]. Viruses such as herpesviruses, retroviruses and papillomaviruses are commonly found in the oral microbiota. Viruses such as Epstein–Barr virus, herpes simplex virus, HIV or hepatitis C viruses serve as reservoir for pathogenic gene functions [30,32]. SARS-CoV-2 was also found in the saliva, and inflammatory- type oral dysbiosis (including microbial species such as *Veillonella* and *Prevotella*) was associated with long COVID-19 [33].

### 3.2. Factors That Can Influence the Oral Microbiome

The oral microbiome characteristics are influenced by genetic and environmental factors [5,34]. The diversity and abundance of the microbiome increase from newborn period to senescence [5] (Figure 1). Variations in sex hormones may influence subgingival microbiome and periodontal disease [35]. The ecosystem’s composition and characteristics are related to several factors such as age, diet, poor oral hygiene, smoking and even dental materials of restorations, crowns, bridges and implants and also prosthetic devices, systemic diseases or medications [36,37,38,39,40,41,42,43,44]. Several host factors can negatively influence the composition of the oral microbiome to a dysbiotic state, altering the balance between the host microbiome toward a harmful relationship [5,27]. 

The transition in diet during human evolution led to a less diverse microbiota and a higher number of microbes associated with tooth decay and periodontal disease [36]. The diet can have habitat-specific effects on oral microbiome [45]. The refined sugar in the diet led to selection of *Streptococcus mutans* to outnumber other oral bacteria [46,47,48,49]. Diet is influencing periodontal diseases as well, as deficiencies in micronutrients (vitamins C, D), antioxidants, low docosahexaenoic acid and low magnesium and calcium serum values correlate with periodontal disorders [50]. Commensal bacteria have an advantage over pathogen bacteria when the host diet is not rich in fermentable carbohydrates [8]. 

Besides other risk factors such as age, genetics, gender, diet and oral hygiene [51], smoking was shown to determine changes in the oral microbiome [52] and also have an immunosuppressant effect [53]. Smoking results in a higher taxonomic diversity and richness in the subgingival plaque, with increased anaerobes such as *Fusobacterium nucleatum*. By contrast, the commensal *Streptococci*, *Granulicatella* and *Actinomycetes* are reduced in smokers’ saliva [30].

## 4. Biofilms in Oral Microbiome

### 4.1. Definition

Biofilms are highly-organized aggregates of microorganisms into an extracellular matrix, frequently self-produced [8,54,55]. Bacteria in biofilms have a cell-to-cell contact, a synergistic lifestyle and a set of unique characteristics different from the free-living cells [55]. 

The oral microbiome forms a biofilm that covers the oral structures, containing a high number of microbes influenced by the composition and surface characteristics they accumulate onto [56,57,58]. The self-produced extracellular polymeric matrix of the biofilm is mainly composed of polysaccharides, proteins, lipids and extracellular DNA [55]. Biofilm formation starts from the salivary pellicle that favors bacterial adhesion, colonization, proliferation and then co-aggregation, followed by the biofilm maturation in a complex microbial community [57]. According to the type of biofilm, bacterial gene expression changes can also occur [55,58].

The in vitro models of the plaque biofilm allowed the testing of antimicrobial or microbe modulating compounds and exploration of biochemical and metabolic interactions among different species and taxa from the oral cavity. Nevertheless, the plaques reconstituted in vitro are not similar to the oral cavity plaque with respect to the spatial structure [59]. Moreover, most studies are performed on single-species populations, whereas recent studies demonstrate the existence of co-operative behavior (for instance, a biofilm between *P. aeruginosa*, *Pseudomonas protegens* and *Klebsiella pneumoniae* synergistically degraded a toxin, which none of the monospecies biofilm was able to degrade) [55]. 

There is an increasingly known exchange of information between microbial communities in the oral microbiome, as well as with the host [60]. *Streptococci* and several other species are important for the spatial and temporal development of oral biofilms and its maintenance through several mechanisms, including the production of hydrogen peroxide (H_2_O_2_), also a signaling molecule [60]. H_2_O_2_ is produced by commensal flora, mainly by *S. sanguis*, and depends on bacterial enzymes such as pyruvate oxidase SpxB, lactate oxidase or L-aminoacid oxidase [60]. Tipping the balance to increase H_2_O_2_ production improves the oral health [60]. 

The self-produced extracellular polymeric matrix of the biofilm is mainly composed of polysaccharides, proteins, lipids and extracellular DNA [55]. The exposure to the toxic ingredients in smokers may relate to oral dysbiosis through immunosuppression, hypoxia or formation and colonization of biofilms with respiratory pathogens such as *Haemophilus* and *Pseudomonas* [30].

### 4.2. Biofilms and Curli Amyloid

Amyloids are proteins with conserved beta sheet structures, which may be produced by host cells and accumulate in tissues in various inflammatory or degenerative diseases. Amyloid fibers called curli may also be produced by bacteria containing the csg gene cluster, as a component of bacterial biofilms, and up to 40% of bacterial biofilms contain amyloids [12]. Curli fibers are produced by *Enterobacteriaceae*, *Bacteroidetes*, *Proteobacteria*, *Firmicutes* and *Theromosulfobacteria* [12]. Curli fibers are involved in cytokine production such as type I interferons, activate the Toll-like receptors TLR1 and TLR2 and the intracellular NLR family Pyrin Domain Containing 3 (NLRP3) inflammasome resulting in inflammation [12,61,62]. Amyloid-producing bacteria have been described in systemic lupus erythematosus, reactive arthritis, neurodegenerative diseases, colorectal cancers and other diseases, and progress in understanding their contribution to disease pathogenesis will hopefully bring about potential therapies [62].

Effective control of oral biofilms is challenging, as microorganisms in biofilms have increased drug tolerance [54]. Several strategies including antimicrobial dental materials based upon antimicrobial agent release, contact killing and combined strategies have been developed lately [54]. More bacteria accumulate on rough than on smooth surfaces of dental materials: on ceramics the biofilms are thin and highly viable, whereas on composites and glass-ionomer the cements cause surface deterioration, which enhances biofilm formation again [39]. Removable dentures favor the accumulation of microbes and biofilm growth by increasing the total surface available, their influence depending on the individual characteristics of the materials [31]. 

In a professional environment, antimicrobial photodynamic therapy has recently emerged as an alternative to mechanical removal of biofilms [54].

## 5. Oral Microbiome in Oral and Systemic Pathology

### 5.1. Oral Microbiome and Periodontitis

**Figure 4:**
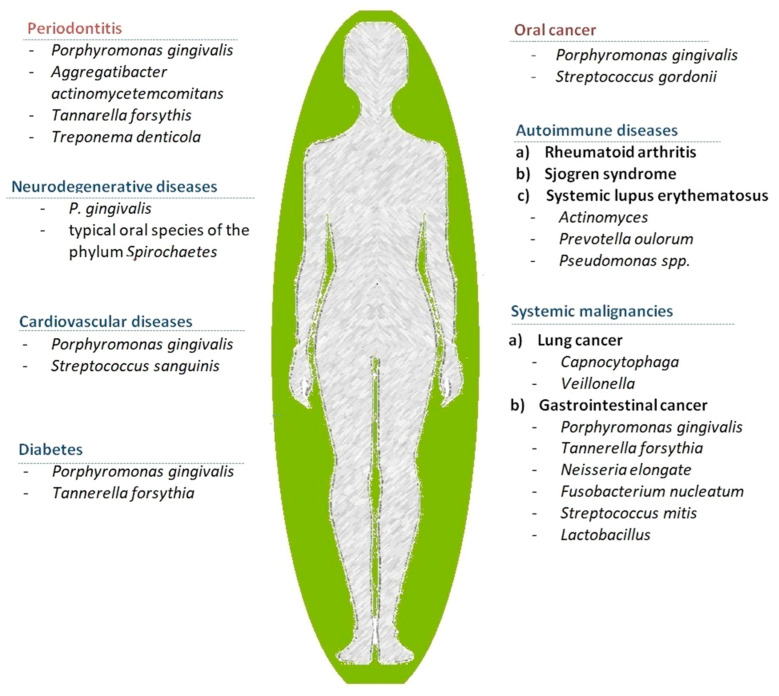
Types of main oral and main systemic diseases related to dysbiosis of the oral microbiome adapted after [6,30,43,44,63,64,65,69].

Disturbances of the symbiotic relation between the host and the oral microbiome may cause oral and systemic diseases [63,64] (Figure 4). Microbes from oral biofilm can spread in other parts of the body through the respiratory or blood systems or to the digestive tract [65]. Periodontitis (PD) is a bacterially-triggered chronic periodontal inflammation resulting in progressive, irreversible destruction of the connective periodontal attachment and alveolar bone resorption and tooth loss [66]. The common forms of PD are associated to anaerobic, Gram-negative bacteria—bacteroides such as *Porphyromonas gingivalis* and *Prevotella intermedia* and spirochetes such as Treponema denticola [66,67,68]. A higher prevalence of *Actynobacillus Actinomycetemcomitans* is characteristic for the localized juvenile PD [68]. 

Oral diseases such as caries and PD are associated, besides infectious diseases such as endocarditis, with systemic diseases and have similar pathways with PD including cardiovascular, neurodegenerative, respiratory or autoimmune diseases, osteoporosis, diabetes, cancer or preterm birth [6,42,43,61,67,69,70] (Figure 4). 

The improvement in periodontal status may parallel the improvement in systemic diseases and, the reverse, the treatment of these diseases could alleviate PD [67]. Early diagnostic and treatment of PD may have an important contribution in systemic disease treatment.

PD was shown to be a risk factor for atherosclerotic cardiovascular disease and thromboembolic events, by permitting the entrance of specific bacteria in the blood stream and thus initiating the inflammatory response of the host. Associations with other factors such as smoking, genetic factors and environmental pollutants can influence the disease progression [71,72].

### 5.2. Oral Microbiome and Diabetes Mellitus

Diabetes mellitus has a bidirectional relation: changes in the oral flora favor diabetes mellitus onset and progression, while high glycemic values alter the microbiome composition [73,74]. Periodontal therapies may improve blood glucose level and metabolic control [75,76,77,78,79,80]. Explanation for the predilection to PD in diabetes mellitus are altered neutrophil function and possibly the formation of advanced glycation endproducts (AGE) and consecutive upregulation of their receptors (RAGE), leading to increased proinflammatory cytokines production [67].

### 5.3. Oral Microbiome and Pulmonary Diseases

Oral bacteria may influence the respiratory pathogens colonization, and chronic obstructive pulmonary disease and pneumonia could be associated to poor oral health and PD [81,82]. Oral pathogens have also been found in broncho-alveolar fluid from patients with cystic fibrosis and improving the oral taxa composition in favor of the normal H_2_O_2_-producing *Streptococcal* commensals could interfere with *Pseudomonas aeruginosa* in this setting [60].

### 5.4. Oral Microbiome and Osteoporosis

Osteoporosis and PD share risk factors such as age, smoking and/or alcohol consumption, body mass index and menopause [66]. Overexpression of inflammatory cytokines may result in the vicious circle of osteoclasts activation, gingival bone resorption, increased periodontal space, bacterial proliferation and inflammation [66].

### 5.5. Oral Dysbiosis and Cancer

Epidemiological studies found associations between oral dysbiosis and cancers. In particular, anaerobic bacteria associated with poor oral health and PD, such as *Fusobacterium nucleatum* and *Porphyromonas gingivalis*, can play a role in oral and cervical tumorigenesis [43,83,84]. Other oral pathogens associated with carcinogenesis are aerobic bacteria such as *Parvimonas*, human papilloma virus in oral and cervical cancers, fungi and parasites [25,31,32,85,86]. The mechanisms of tumorigenesis related to oral microbiome, mainly in head and neck cancers, are multiple: suppression of the protective immune response, synthesis of mutagens such as aldehydes, bacterial cytotoxins promoting DNA damage or chronic inflammation [84,87]. 

Periodontal bacterial infections increase cancer incidence, poor survival, disease-free survival and cancer-specific survival. In this instance, *P. gingivalis* and *Prevotella intermedia* increased cancer risk, unlike *Tannerela forsytihia*, *Treponema denticola*, *F. nucleatum* or *Aggregatibacter actinomycetemcomitans* [87,88]. 

Some commensals in the oral cavity may be related to distant cancers: individual number variations (increase or decrease), certain organisms as predictors or changes in different indexes may function as biomarkers [87]. For instance, *P. gingivalis* and *Fusobacterium* increased in oral rinse were associated with different types of cancer in several studies [87]. The combination of *Neisseria elongata* and *Streptococcus mitis* was described as suggestive for pancreatic cancer [89]. On the contrary, some species may be protective for certain cancers, such as *Neisseria* in esophageal cancer, possibly by activation of carotenoid biosynthesis pathway [30,90].

### 5.6. Oral Dysbiosis and Autoimmune Diseases

Environmental and microbial interactions at mucosal sights could trigger autoimmunity in genetically susceptible hosts [30,91]. Gastrointestinal tract dysbiosis (microbiota composition changes, loss of beneficial with relative growth of harmful microorganisms, loss of microbial diversity) contributes to tolerance loss resulting in development of immune rheumatic diseases [92]. Oral dysbiosis could also lead to autoimmunity through multiple mechanisms including autoantigens overproduction, microbial translocation, molecular mimicry, superantigens, checkpoints dysregulation, bystander activation, TLRs dysregulation, cytokines hyperproduction, epitope spreading and autoantigens complementarity [reviewed by 61]. Genetic factors may influence the microbial–host interactions, as HLA-DR4 could enhance innate immune responses after bacterial challenge, while HLA-DQ8 could favor antigen-specific autoreactivity [91].

The development of rheumatoid arthritis (RA) may be influenced by pathogenic bacteria overgrowth, lack of immune-modulating commensal bacteria or long-lasting epigenetic changes in the synovial antigen presenting cells or in the stem cells induced by danger signals (“trained immunity”) [93]. Moreover, commensal bacteria may turn opportunistic in the oral cavity, leading to oral or systemic pathology [92,94]. Periodontal bacteria induce the neutrophil, monocyte and T and B responses with proteinases, cytokines and prostaglandins, with bone resorption similar to RA [94]. Moreover, oral microbiome profoundly influences the gut microbiome [92,95]. *Lactobacillus*, belonging to the *Firmicutes* phylum, was generally associated with anti-inflammatory activity, but its role has been regarded as controversial lately [92]. Moreover, oral bacteria DNA was found in the synovial fluid of patients with RA [96].

In rheumatoid arthritis (RA), PD is more frequent than in controls, and periodontal disease and RA share multiple risk factors, such as smoking and genetic association with HLA-DR [91]. Protein citrullination thus rendering them antigenic is involved in the disease pathogenesis, and the anticitrullinated protein antibodies (ACPA) are a disease marker. Bacteria containing the enzyme peptidylarginine deiminase (PAD) are involved in the citrullination and generation of neoantigens at mucosal sites. *Prevotella intermedia*, *Tanerella forsythia*, *Treponema denticola* and *Porphromonas gingivalis*, main bacteria involved in periodontal disease, possess PAD [97]. Moreover, anti-*P. gingivalis* antibodies are associated with ACPA in individuals at risk [98]. Nevertheless, the role of *P. gingivalis* in RA has been challenged in already-installed RA [95,99]. Moreover, *Agreggatibacter actinomycetemcomitans* is also a trigger for RA as it secretes leucotoxin A which contributes to neutrophil extracellular traps (NETs) generation and release of citrullinated antigens [99,100]. Microbiome switch in oral cavity might contribute to the disease pathogenesis in RA; in preclinical high-risk individuals, microbial diversity and richness was reduced compared to established RA and healthy controls [99,101]. PD is associated with RA severity and ACPA positivity [102]. Moreover, the abundance of *Prevotella* spp., associated with PD, along with a decrease in the normally present *Streptococcus* and *Rothia* spp., is associated with arthritis worsening and production of inflammatory mediators including interleukin-17, tumor necrosis factor-alpha (TNFα) and interferon gamma [103,104]. However, no unique oral bacterial cluster has been demonstrated to be associated with RA so far [104].

In juvenile idiopathic arthritis (JIA), some species such as *Solobacterium* and *Mogibacterium* were found enriched in saliva [105]. 

In Sjogren’s syndrome (SSj), a systemic autoimmune disease evolving with lymphocytic infiltration of exocrine glands, resulting hyposalivation and xerostomia are associated with increased cervical caries incidence, increased *Candida*, *S. mutans* and *Lactobacillus* species colonization [106,107]. Nevertheless, oral dysbiosis in SSj can occur independent of hyposalivation, with a lower oral microbial species diversity [108]. Molecular mimicry could play an important role in SSj pathogenesis, as some oral commensals such as *Campnocytophaga ochraceaea* along with other gut or skin bacteria contain peptides that can activate T cells reactive to Ro60, an autoantigen in SSj and systemic lupus erythematosus (SLE), in order to activate the B cells [109]. 

In systemic lupus erythematosus, oral ulcers are frequent and innate immunity is activated, as increased type I interferons, required for cell apoptosis and pathogen clearance, are associated with disease activity [110]. Moreover, periodontal inflammation was more frequent in SLE, associated with *Fretibacterium*, *Prevotella nigrescens* and *Selenomonas* spp. [111]. 

In systemic sclerosis, a disease evolving with cutaneous infiltration and thickening, the reduced oral aperture and the alveolar bone resorption that may occur in the disease also influence the local biomechanics and the microbial populations. The *Lactobacillus* spp. is reduced significantly on the tongue and in the oral cavity of systemic sclerosis patients [92]. Moreover, *Lactobacilli* are reduced in the diffuse form of systemic sclerosis with respect to the limited form of the disease [92]. 

In ankylosing spondylitis (AS), oral ulcers are more frequent than in the general population [112]. Moreover, in AS, antibodies against *Porphyromonas gingivalis* and *Prevotella intermedia*, respectively, were found in higher titer than in healthy subjects [97]. The saliva of AS patients was found to be enriched with *Brucella* spp. and *Campylobacter concisus*, in *Clostridia* such as *Veillonellaceae*, and depleted of *Bacilli* such as *Streptococcus* [113]. However other studies in AS did not find evidence of any single taxa associated with axial spondylarthritis in the subgingival plaque [114]. 

In Behçet’s disease, *Streptococcus* species have been described in the oral cavity, on mucosa and in saliva and dental plaque. *S. mutans* in patients with severe disease is associated with low levels of mannose-binding lectin, a host defense protein [115]. Moreover, the salivary microbiome reveals an increase in colonization with *S. salivarious* and *S. sanguis* in ulcer sites [115]. The microbial population is different at the ulceration sites with respect to other oral locations [116,117] (Table 1).

Oral microbioma may generally be influenced by the therapy of systemic autoimmune diseases. In RA, the disease-modifying drug therapy partially restored a normal oral microbiome, including increase in *Prevotella* spp., more abundantly found in healthy controls, and reduction in *Veillonella* [95]. The oral microbial signature in RA before and after methotrexate could predict the response to therapy [95]. Antibiotics may improve oral microbiome but may nevertheless induce gut dysbiosis and not improve arthritis in RA [93]. By contrast, periodontal therapy may alleviate disease activity in RA [122]. 

In juvenile idiopathic arthritis, no difference was found in the patients treated with biologics alone or in combination with methotrexate with respect to the microbiota [105].

In AS, sulfasalazine (an immunomodulatory drug with antibiotic properties) is effective in the peripheral form of disease [97]. In AS, the anti-TNF therapy improved the periodontal status along with the AS disease activity parameters, possibly suggesting an effect on the periodontium ligament [123]. 

In Behçet’s disease, besides local therapies including regular oral hygiene and topical therapies such as mouthwashes, antibiotics such as macrolides (azithromycin) also have immunomodulatory effects, decreasing the interferon responses to *S. Sanguis* [115]. Nevertheless, immunosuppression (cyclosporine A, azathioprine and prednisone) did not modify the modified microbioma, which was influenced by the PD therapy instead [120].

The salivary microbioma composition depends on the circadian rhythm, some genera showing significant periodicity, linking the oral microbiome with the salivary cytokine [124]. *Prevotella* was most significantly associated with diurnal variations of interleukins IL-1β (and to some extent to IL-6 and IL-8) [124]. However, the time of meals does not seem to influence the oral inflammatory and metabolic biomarkers [125].

Patients’ stratification and microbiome-based therapy in patients at risk or with an established autoimmune disease could be of great interest for the lifelong management of autoimmune diseases [104,113]. Variations in oral microbial species may differentiate rheumatoid arthritis from osteoarthritis, and eight bacterial biomarkers (*Actinomyces*, *Neisseria*, *Neisseria subflava*, *Hemophilus parainfluenzae*, *Hemophilus*, *Veillonella dispar*, *Prevotella* and *Veillonella*) were selected in the prediction model to help distinguish between RA and OA [126]. 

Oral health care, especially of microbiota, should receive attention in the daily health care of autoimmune disease patients, as the altered salivary microbiota and their metabolites may influence the disease flares and severity [93,113]. The role of probiotic supplementation in autoimmune diseases in general is controversial and requires tailored strategies which have to be proved efficient [92].

In the COVID-19 era, the widespread use of disinfectants could alter microbial diversity and load, favoring autoimmunity; moreover, quaternary ammonium compounds may impair innate immune cell function, raining concerns of a future development of autoimmune disease [127]. Parkinson’s and Alzheimer’s diseases have been reported as neurodegenerative disorders associated with peculiarities of oral dysbiosis. Animal studies (in mice) have shown that *P. gingivalis* infection gave brain colonization, and enzymes produced by *P. gingivalis* have neurotoxic effects. Moreover, an association between *P. gingivalis* and Alzheimer’s disease was reported [6,30]. Other microorganisms, such as *Prevotella*, *Fusobacteria* and *Actinomyces*, have been found in Alzheimer’s disease and in the periodontal pockets [67]. It has also been shown that typical oral species of the phylum *Spirochaetes* (including multiple species of the genus *Treponema*) often comprise amyloid plaques [6,30]. The curli-producing bacteria may also be involved, due to structural similarities between curli and human amyloids such as β-amyloid involved in Alzheimer’s disease, α-synuclein in Parkinson’s disease and serum amyloid A [128]. The molecular mechanisms of their action by oral taxa make this field an attractive area of research [6,30].

## 6. How to Influence the Oral Microbiome

Periodontal therapy has been shown to influence the disease activity or the biomarkers in several systemic diseases [67]. Hygiene habits influence the oral biofilm formation. Poor hygiene favors bacterial accumulation, as the salivary pellicle forms within seconds after cleaning [129]. Oral hygiene is also a key factor to prevent systemic diseases caused by the spreading of the microbes to different parts of the body [130]. Mechanical oral hygiene using dentifrice and toothbrush ensures the dental plaque removal. Mouth rinses may supplement oral hygiene and are useful in gingivitis or periodontal diseases [131]. The ingredients of dentifrices and mouth rinses influence the oral microbiome composition. 

### 6.1. Antimicrobials

Antimicrobials, both of synthetic and natural origin, are used mainly for anti-caries benefit, being the agents with the greatest influence on the oral microbiome. 

Chlorhexidine (CHX) remains the most common antimicrobial agent widely used in oral care products for its broad-spectrum and long-lasting antibacterial activity. CHX reduces the proliferation of several bacterial species linked to caries (such as *Streptococcus mutans*), linked to PD such as *Actinomyces*, *Porphyromonas gingivalis*, *Enterobacteria*, *Fusobacterium nucleatum* or to halitosis-related bacteria such as *Porphyromonas gingivalis*, *Enterococcus faecalis* [132,133]. The use of CHX increases favorable bacterial families such as *Streptococcaceae*, *Carnobacteriaceae*, *Neisseriaceae* and *Flavobacteriaceae* [132,134]. In the meantime, CHx use decreases *Prevotellaceae*, *Clostridiaceae*, *Fusobacteriaceae*, *Lachnospiraceae*, *Campylobacteraceae*, *Actinomycetaceae* and *Corynebacteriaceae* [132,134]. 

Cetylpiridinium chloride (CPC) 0.05% in mouth rinses exhibits antimicrobial activity against periodontal pathogens. Thus, at concentrations of 0.3–0.7%, it inhibits *Actinomyces*, *Campylobacter*, *Moraxella*, *Veillonella*, *Eikenella corrodens*, *Porphyromonas gingivalis* and *Prevotellae*, while at concentration of 6%, other microorganisms such as *Aggregatibacter*, *Candida* and *Streptococci* were inhibited [135]. Nevertheless, the long-term use of CHX and CPC with sub-lethal concentrations was associated with bacterial resistance (reported for *Streptococcus mutans*, *Streptococcus sobrinus*, *Porphyromonas gingivalis*, *Fusobacterium nucleatum*, *Prevotella intermedia* and *Aggregatibacter actinomycetemcomitans*) [136].

Sodium hypochlorite 0.05%, octenidine dihydrochloride 0.1% and povidone iodine 10% were proved to be able to reduce the vitality of periodontal pathogens such as: *Porphyromonas gingivalis*, *Aggregatibacter actinomycetemcomitans*, *Fusobacterium nucleatum* and others [137].

### 6.2. Prebiotics

Prebiotics are non-viable food components that confer a benefit to the host associated with modulation of the microbiota. Arginine is an amino acid functioning as a prebiotic, which may also influence the oral ecosystem. Arginine can destabilize the oral biofilm, also decreasing the dentinal hypersensitivity and of the enamel demineralization [138,139]. Oral bacteria have an arginine deaminase system which metabolizes arginine, thus increasing the pH of the oral cavity [140]. Toothpastes with arginine (8%) significantly increased *Veillonella* in saliva [141]. L-arginine monohydrochloride increases *Streptococcus* and *Veillonella* and decreased *Neisseria* and *Aggregatibacter* in the oral biofilm [139]. 

D-tagatose, a non-cariogenic sugar abundant in the saliva of individuals with good oral health, suppresses growth of *S. mutans* and causes species-specific transcriptomic and metabolomic changes in *S. mutans*, *S. gordonii* and *S. oralis* [11].

### 6.3. Probiotics

The oral microbiota is influenced also by the consumption of probiotics, living microorganisms with safety profile for human ingestion which provide health benefits when they are present in specific concentrations. *Lactobacillis decreases* the number of *S. mutans* related to caries [142]. Some strains can act directly on pathogenic bacteria: *Lactobacillus reuteri* by producing molecules with antimicrobial activity and vitamins B_12_ and B_6_, *Bifidobacterium bifidum* and some species of *Lactobacillus* (*L. johnsonii*, *L. crispatus*, *L. jensenii*) are able to produce hydrogen peroxide which can act on the epithelium of other bacteria causing their death [143], *L. casei* produces biosurfactants which act on the preformed biofilms by dispersing them, *L. acidophilus* produces lipases which degrade the biofilm, while the secretory factors of *L. salivarius* reduces the formation of biofilm and also the pathogenicity of *Candida albicans* [144]. Moreover, probiotics such as *Lactobacilli* may attach to the mucus lining, selectively interact with host immunocompetent cells such as dendritic cells and improve the epithelial barrier function. Overall, probiotics may help control dysbiosis and alleviate local and systemic inflammation [145]. 

### 6.4. Postbiotics

Postbiotics are the microbiome-derived metabolites found in high concentration throughout the digestive system and also in the systemic circulation [34,59]. Immunomodulatory effects of postbiotics isolated from *Bacillus coagulans* or *Bifidobacterium breve* induce anti-inflammatory cell responses, and products containing *L. paracasei* postbiotic reduce incidence of pharyngitis [10]. Therapeutic strategies using postbiotics to modulate oral microbiome, mainly in infants, are promising directions of development [10]. 

### 6.5. Surface Active Agents

Surface active agents, such as delmopinol hydrochloride, impede the synthesis of glucan polysaccharide, a natural bioadhesive which promotes the formation of biofilm matrix and its adhesion [146]. Sodium lauryl sulfate interacts with the lipids and the proteins of the bacterial cell membrane exhibiting thus antimicrobial activity. Due to its deep penetration into oral biofilms, it was shown that it is able to inhibit plaque formation and has strong antimicrobial activity against *Streptococcus mutans* at concentrations ranging between 1.0 and 1.5%, at which it is usually present in dentifrices [147,148]. 

### 6.6. Abrasive Agents

Sodium bicarbonate, an abrasive agent used in dentifrices, influences the composition of oral microbiota by its pH modulating ability and its antimicrobial activity. At concentrations from 75 µM/L to 100 mM/L, it acts as bactericidal for several microorganisms from oral biofilm such as *Haemophilus aphrophilus*, *Capnocytophaga gingivalis*, *Actinobacillus actinomycetemcomitans* and *Eikenella corrodens*, and at concentrations between 52 and 65%, it decreases the level of *Fusobacterium*, *Actinomyces* sp. and *S. mutans* [149]. Other abrasive agents, tetrasodium and/or tetrapotassium pyrophosphate and polyphosphates, also exert antimicrobial activity. Dentifrices with tetrasodium pyrophosphate decrease *Spirochetes* (including *Treponemae genera*—*T. denticola*, *T. vincentti*, highly pathogenic at periodontal level), *Proteobacteria* and *Fusobacteria* while increasing *Streptococcus genera* associated with oral health [150]. 

### 6.7. Plant-Derived Ingredients

Many oral hygiene products contain extracts from plants with antimicrobial properties as an alternative to synthetic antibacterial which has several side effects and may contribute to antimicrobial resistance (Table 2).

The extracts prepared from the leaves of the plant *Camellia sinensis* are known for their multiple biological activities: anti-inflammatory, antimicrobial, antioxidant, anti-carcinogenic and anti-allergic [151]. More specific teaflavins (Tfs), the principal active ingredients from black tea, were shown to have antibacterial activity against *S. mutans* [152] and *Porphyromonas gingivalis* [153]. Teaflavins toothpastes reduce the bacteria responsible for various oral pathologies, while not influencing the bacteria related to oral health [154]. 

Essential oils obtained from Melaleuca alternifolia, Melissa officinalis and Lavandula angustifolia were shown to have antibacterial activity against *Staphylococcus aureus* and *Escherichia coli* from oral biofilms [156]. *Origanum vulgare, Thymus vulgaris* and Eugenia caryophyllata essential oils were proven efficient against *Actinomyces viscosus*, *Enterococcus faecalis*, *Streptococcus oralis*, *S. sanguinis*, *S. salivarius* and *S. mutans* [157]. *Cymbopogon nardus* essential oil was shown to be efficient against *Candida albicans* and *Staphylococcus aureus* biofilms from prosthetic materials [158], and Syzygium aromaticum and several other essential oils were reported efficient against *Porphyromonas gingivalis* and *Fusobacterium nucleatum*, both related to periodontal diseases and halitosis [159,160,161,162] (Table 2).

### 6.8. Other Ingredients

Enzymes and proteins present in toothpastes modified the composition of the oral microflora, increasing the bacterial species associated with oral health: *Prevotella melaninogenica*, *Neisseria* sp., *Granulicatella elegans* and *Lactobacillus gasseri*, a potential probiotic with antibacterial activity against *Porphyromonas gingivalis*. Enzymes and protein inhibited the growth of microorganisms known to be related to periodontal diseases: *Treponema*, *Fusobacterium*, *Prevotella* (*P. intermedia*) and *Eubacterium* related to PD [163]. 

## 7. Oral Microbiome and Personalized Medicine

The characteristics of the oral microbiome are highly important for a complete understanding of the interactions with the host and also for the possibility of diseases prevention, diagnosis and treatment. The existing specific microbiome for each individual has an essential contribution in the onset of the disease, as disease may develop differently among different individuals [19]. The patient’s microbial profile may serve as biomarkers for evaluating the risk for disease, for early treatment, assessing response to therapy or to guide new treatments and prophylactic measures [16,29,164,165]. Research on the microbiome and its genomes in two important fields, microbiomics and metagenomics, will not only contribute to identifying the individual microbial profile but also in discovering their functions and interaction with the host [29，^164^]. Of interest, several studies on neonates have discussed the role that microbiota, which is altered in infants born through cesarean section, may play in early stress reactivity [25,166,167]. Knowledge on metagenome sequencing could be necessary in the future for medical practitioners for *on-the-spot* microbial identification and specific intervention as part of personalized medicine [25].

## 8. Conclusions and Perspectives

The oral microbiome is an expanding field of evaluation and research. The oral biofilm is a biological target in prevention and strategies for modulation in health and disease. A good oral hygiene and a smooth surface of dental restorations and prosthetic devices, as well as antimicrobial materials could contribute to a thinner biofilm, reducing bacterial growth and adhesion. 

The progress in the extracellular matrix properties research contributes to a better understanding of the bacterial biofilm, besides the genetic and metabolic pathways [55]. Exploring the functions of the commensal oral or general microbiome and interactions with immune system, with implications in health and diseases, requires more studies. The effects of the interaction between the microbiome, virome and mycobiome add a layer of complexity in understanding their impacts on innate and adaptive immune responses. Integration of multi-omics data, including epigenomics, will help in clarifying the mechanisms that explain the high cross regulation of the oral microbiome and immune system. A successful translation of microbiome-based approaches into clinical practice needs unbiased and standardized preclinical and clinical studies. Collaboration between stomatologists, microbiologists, geneticists, pharmacists and other specialists, focused on the oral microbiome and the oral biofilm, is increasingly employed in the caring of patients with systemic diseases. Oral microbiome analysis will emerge as a new approach in prevention of systemic diseases. Future personalized therapies for oral and systemic diseases should also aim to restore the normal symbiotic relation with the oral microbiome.

## Figures and Tables

**Table 1:** Oral microbioma in several autoimmune diseases.

Disease	Microbiota Changes	References
Rheumatoid arthritis	*Veillonella* increased in saliva of RA; *Lactobacillus salivarius* overrepresented in saliva, mostly in very active disease; *Atopobium* spp., *Cryptobacillum curtum* enriched in saliva and dental plaque *Rothia mucilaginosa*-like enriched in dental plaque and saliva; *R. dentocariosa* enriched in dental plaque *Butyrivibrio* spp., *Atopobium parvulum*, *Prevotella* spp., *Solobaterium moorei*, *Centipeda* sp., *Veillonella* spp., in RA *Anaeroglobus geminatus* correlated with ACPA and rheumatoid factor in saliva *Hemophilus* spp. depleted	[95,101,102]
Juvenile idiopathic arthritis	*Solobacterium*, *Mogibacterium* and *TM7-G1* enriched in saliva	[105]
Ankylosing spondylitis	*Brucella* spp. and *Campylobacter concisus*, *Clostridia* such as *Veillonellaceae* increased in saliva *Streptococcus* depleted in saliva	[97,113]
	The saliva of AS patients enriched in *Veillonella* spp., *Brucella* spp., *Campylobacter concisus* and depleted in *Streptococcus* spp.	[113]
Sjogren’s syndrome	*Lactobacillus* spp., *S. mutans, Candida albicans* increased in supragingival plaque *Fusobacterium* decreases on the tongue *Capnocytophaga ochracea* derived microbial peptides can activate Ro60-reactive T cells	[109,118]
Systemic lupus erythematosus	*Fretibacterium, Prevotella nigrescens, Selenomonas* spp. are increased, associated with local release of IL-6, IL-17, IL-33 *Lactobacillae*, *Veillonaceae* and *Moraxellaceae* increased, while *Corynebacteriaceae*, *Micrococcaceae*, *Sphingomonadaceae*, *Halomonadaceae* and *Xanthomonadaceae* decreased	[111,119]
Systemic scle rosis	*Lactobacillus* spp. are reduced significantly on the tongue, the oral mucosa, mainly in the diffuse form of disease	[92]
Behçet’s disease	*Streptococcus* spp. increased on oral mucosa, in saliva and dental plaque, *Rothia dentocariosa* increased in non-ulcer sites *Hemophilus parainfluenzae* increased in saliva *Bifidobacter dentium*, *Prevotella histicola*, *Candida albicans* increased in saliva in active disease *Alloprevotella rava*, *Campylobacter concisus*, *Clostridiales* spp. *Neisseria* spp. depleted in the saliva	[116,117,120]
Henoch-Schön lein purpura	Higher oral microbial diversity and richness, with dominance of *Firmicutes*, *Proteobacteria* and *Bacteroidetes*	[121]

**Table 2:** Plant-derived ingredients that influence the oral microbiome.

Plant Extracts	Plants of Essential Oils	Biological Activities on Oral Microbiome		References
*Camellia sinensis*		Anti-inflammatory, antimicrobial, antioxidant, anti-carcinogenic and anti-allergic		[151]
Black tea (teaflavins)		Antibacterial activity against *S. mutans* and *Porphyromonas gingivalis*		[152,153]
		Decreases bacteria responsible for various oral pathologies		[154]
*Melaleuca alternifolia* and propolis		Eradication of *Candidate albicans*, *Campylobacter gracilis*		[155]
		Reduces *Streptococcus mitis* and *Streptococcus sanguis*		[155]
	*Melaleuca alternifolia*, *Melissa officinalis* and *Lavandula angustifolia*	Antibacterial activity against *Staphylococcus aureus* and *Escherichia coli* from oral biofilms		[156]
	*Origanum vulgare*, *Thymus vulgaris* and *Eugenia caryophyllata*	Efficient against *Actinomyces viscosus*, *Enterococcus faecalis*, *Streptococcus oralis*, *S. sanguinis*, *S. salivarius* and *S. mutans*		[157]
	*Cymbopogon nardus*	Efficient against *Candida albicans* and *Staphylococcus aureus* biofilms from prosthetic materials		[158]
	*Syzygium aromaticum*	Good activity against *Porphyromonas gingivalis*		[159]
	*Citrus aurantium*	Antimicrobial activity against *S. mutans,* significant decrease in several virulent genes expressed by *S. mutans*		[159]
	Cinnamomum verum (oil from barks)	Efficient against *S. mutans*		[160]
	*Cinamomum verum bark*, *Cinnamomum zeylanicum*, *Origanum majorana*, and *Melaleuca alternifolia*	Efficient against *Solobacterium moorei*	oral bacteria responsible for halitosis	[161,162]
	*Aloysia gratissima*, *Aloysia triphylla*, *Alpinia speciose*, *Artemisia capillaris*, *Baccharis dracunculifolia*, *Callitris glaucophylla*, *Chrysanthemum indicum*, *Commiphora myrrha*, *Coriandrum sativum*, *Cymbopogon citratus*, *Cyperus articulatus*, *Elyonurus muticus*, *Eugenia caryophyllata*, *Ficus deltoidea*, *Juniperus communis*, *Melaleuca alternifolia* and *Mentha piperita*	Efficient against anaerobic bacteria *Porphyromonas gingivalis* and *Fusobacterium nucleatum*		[162]

## Data Availability

Not applicable.
